# Design of a monolithic cellulose Janus-like membrane for oil/water separation

**DOI:** 10.1186/s40643-026-01142-0

**Published:** 2026-10-09

**Authors:** Mamadou Souare, Changqing Dong, Xiaoying Hu, Junjie Xue, Junjiao Zhang

**Affiliations:** 1 National Engineering Laboratory for Biomass Power Generation Equipment, School of New Energy, North China Electric Power University, Beijing, 102206 China; 2 State Key Laboratory of Alternate Electrical Power System with Renewable Energy Sources, North China Electric Power University, Beijing, 102206 China; 3 School of Energy, Power and Mechanical Engineering, North China Electric Power University, Beijing, 102206 China

**Keywords:** Cellulose acetate, Janus membrane, Oil/water separation, NIPS, multilayer membrane

## Abstract

Janus membranes offer a good solution for oil/water mixtures due to their opposite wettability, but existing designs often suffer from limitations arising from complex, resource-intensive fabrication procedures. This study proposes a cellulose-based monolithic Janus-like membrane prepared by non-solvent-induced phase separation (NIPS) from cellulose acetate followed by alkaline deacetylation and unilateral silanization (TEOS/MTES). The resulting membrane exhibits asymmetric wettability (hydrophobic: 91°, hydrophilic: ~0°). Structural and chemical characterization (SEM, EDS, FTIR, and BET/BJH) confirms the formation of a siloxane-containing surface layer, the integrity of the cellulose network, and a distribution of the pores toward the nanoscale, with a specific surface area of 26.9 m^2^/g. Gravitational peanut oil/water tests reveal high fluxes (3300–5527 Lm^−2^h^− 1^) with an apparent volumetric oil-retention efficiency of up to 95%, while for water/hexane, the apparent volumetric oil-retention efficiency reached 96.93% with a flux of 3950.62 Lm^−2^h^− 1^. The membrane retains excellent mechanical robustness (3.50 MPa), with a breaking force of 5.23 N, withstanding repeated deformations including axial rolling, compression, and bending, without cracking or delamination. *This* Janus-like membrane mimics the properties of Janus membranes while relying on a simplified, energy-efficient, and environmentally friendly fabrication strategy, and could represent a promising option for efficient, sustainable, and high-performance oil/water separation.

**Supplementary Information:**

The online version contains supplementary material available at https://doi.org/10.1186/s40643-026-01142-0.

## Introduction

The contamination of aquatic environments by hydrocarbons remains a major environmental and health issue, fueled by accidental spills, industrial effluents, and oil production water (Tian et al. 2023). These discharges generate complex oil/water mixtures, often in the form of stable, finely dispersed emulsions, the treatment of which represents a considerable technical challenge (Yang et al. 2025). Conventional separation methods, such as gravity separation, flotation, skimming, coagulation, flocculation, or adsorption, often have significant limitations for oil/water mixtures, including high energy consumption, complex equipment, and the generation of secondary pollution (Su et al. 2025, Souare et al. 2024, Xie et al. 2021). In this context, membrane separation technologies have emerged as attractive solutions due to their high efficiency, modularity, and ability to operate continuously while consuming minimal energy (Wu et al. 2024, Xin et al. 2024).

The performance of membranes designed for oil/water separation depends on a delicate balance between permeability, selectivity, and fouling resistance. These characteristics are governed by the membrane’s porous structure, surface chemistry, and interfacial energy (Xin et al. 2024, Huang et al. 2024). Membranes with special wettability have attracted growing interest, as they allow selective control of the affinity of surfaces to water and oil (Su et al. 2025, Hu et al. 2025, Shui et al. 2025). However, many of the superhydrophilic or superhydrophobic membranes reported have fixed surface wettability (Wang et al. 2020, Zong et al. 2025, Zhang et al. 2023, Yang et al. 2023, Wang et al. 2024), which can limit their adaptability to the variety of oil/water mixtures encountered in practical conditions (Yu et al. 2025). To address this limitation, Janus membranes, defined by asymmetric wettability on their two sides, have emerged as a particularly promising strategy (Wang et al. 2024, Afsari et al. 2021). The coexistence of a hydrophilic and a hydrophobic side enables directional and selective fluid transport, thereby improving separation efficiency and resistance to clogging (Kuang et al. 2025, Zhang et al. 2021, Meng et al. 2023, Wang et al. 2025). However, reported fabrication strategies rely on the modification of preformed substrates or the construction of multilayer assemblies (Doguwa et al. 2025, Cao et al. 2025, Lin et al. 2025). These designs are often hampered by limited interfacial adhesion, the risk of delamination under pressure or shear, progressive degradation of the functional layers, and complex manufacturing processes (Lin et al. 2025, Wang et al. 2019). In addition, these approaches require the use of solvents of concern, such as N, N-dimethylformamide (DMF), chloroform, dichloromethane, and dimethylacetamide (DMAc), or fluorinated agents (Li et al. 2025, Kong et al. 2025, Thamaraiselvan and Wickramasinghe 2025, Si et al. 2023), which raises environmental and regulatory concerns.

Thus, the rational design of high-performance Janus membranes for oil-water separation still needs to be further developed, particularly to meet the following challenges:

1. the use of renewable biomass-derived materials as sustainable alternatives to conventional synthetic polymers.
2. the reduction of hazardous chemical solvents to enable safer and more environmentally friendly fabrication processes.
3. the transition from multilayer to monolithic membranes to enhance structural integrity and simplify fabrication.

In this work, we propose the design of a monolithic cellulose-based Janus-like membrane with unilateral interfacial modification to address the identified scientific and technological challenges. A membrane is first developed by non-solvents induced phase inversion (NIPS) from cellulose acetate using an environmentally improved solvent, supplemented with a small fraction of a non-solvent additive and a controlled pre-evaporation step, allowing fine adjustment of phase-separation kinetics and pore morphology. The acetate membrane is then converted to regenerated cellulose (RCM) by alkaline deacetylation, providing an intrinsically hydrophilic and chemically reactive substrate. Finally, selective silanization of a single side is performed to introduce localized hydrophobic functionality, while preserving the opposite side in its native hydrophilic state. This strategy avoids layer stacking, ensures good interfacial integrity, and combines pore formation with unilateral surface chemical modification through sequential processing. The resulting monolithic Janus-like membrane enables directional oil/water separation, in which the hydrophobic side limits oil intrusion and reduces droplet adhesion, while the hydrophilic side promotes continuous water transport. By combining rational porosity control via NIPS with asymmetric surface chemistry achieved through single-sided silanization, this work establishes a robust design framework for high-performance cellulose Janus-like membranes that is compatible with a sustainable approach and can be scaled up to industrial production. This approach decouples the bulk pore structure from surface wettability, thereby overcoming the limitations of multilayer or coated Janus membranes.

## Materials and methods

### Materials

Cellulose acetate (CA, average molecular weight Mn ≈ 53,000 g·mol^− 1^, determined by gel permeation chromatography (GPC); CAS No. 9004-35-7) was purchased from Macklin Biochemical Co., Ltd. (Shanghai, China) and used as the membrane-forming polymer. Dimethyl sulfoxide (DMSO) and acetone were used as solvents for the casting solution, sourced from Macklin Biochemical Co., Ltd. (Shanghai, China). Deionized water served as a non-solvent additive and as the coagulation medium during the non-solvent induced phase separation (NIPS) process. Sodium hydroxide (NaOH) and ethanol were obtained from Macklin Biochemical Co., Ltd. (Shanghai, China) and employed for the alkaline deacetylation of the cellulose acetate membrane to produce regenerated cellulose. Tetraethyl orthosilicate (TEOS) and methyltrimethoxysilane (MTES), used as silane precursors for one-sided hydrophobic surface modification via the sol-gel process, were supplied by Macklin Biochemical Co., Ltd. (Shanghai, China). Deionized water was used for all solution preparations, membrane coagulation baths, washing, and storage procedures. All chemicals were used as received without further purification.

### Preparation of monolithic membrane

**Fig. 1:**
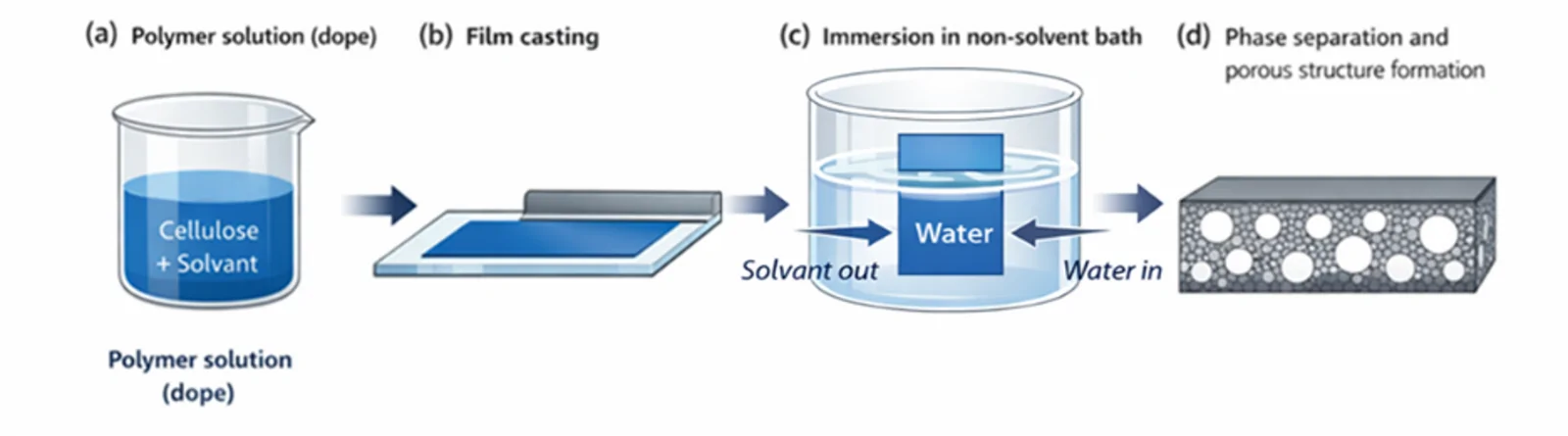
Schematic illustration of membrane fabrication via the NIPS process: **a** including dope solution preparation, **b** casting on a glass substrate, **c** immersion in a coagulation bath, and **d** formation of a porous monolithic membrane

A dope solution was prepared by dissolving 18 g of cellulose acetate in 60 g of dimethyl sulfoxide (DMSO) at 33 °C in a 500 mL beaker under stirring until a homogeneous solution was obtained. Then, 40 g of acetone was slowly added to improve miscibility and ensure complete homogenization, followed by the addition of 2 mL of water to induce non-solvent-induced phase separation (NIPS). The resulting solution was degassed at room temperature for 12 h to remove air bubbles. The membrane was then fabricated using the NIPS method at room temperature, as shown in Fig. 1. The solution was cast onto a glass plate using a doctor blade (200 μm), and the film was exposed to air for 20 s to allow partial solvent evaporation. Phase inversion was initiated by immersing the film in an ultrapure water bath at 25 °C for thirty minutes. The formed membrane was then detached, transferred, and rinsed several times to remove residual solvents.

To obtain regenerated cellulose membranes (RCM), cellulose acetate membranes were deacetylated using alkaline hydrolysis. The membranes were immersed in a 0.5 M NaOH solution prepared in an ethanol: water mixture (70:30, v/v) at 30 °C for 2 h. After deacetylation, the membranes were rinsed with ultrapure water until the pH reached neutrality and stored hydrated for further surface modification.

### Surface modification for Janus-like membrane fabrication

**Fig. 2:**
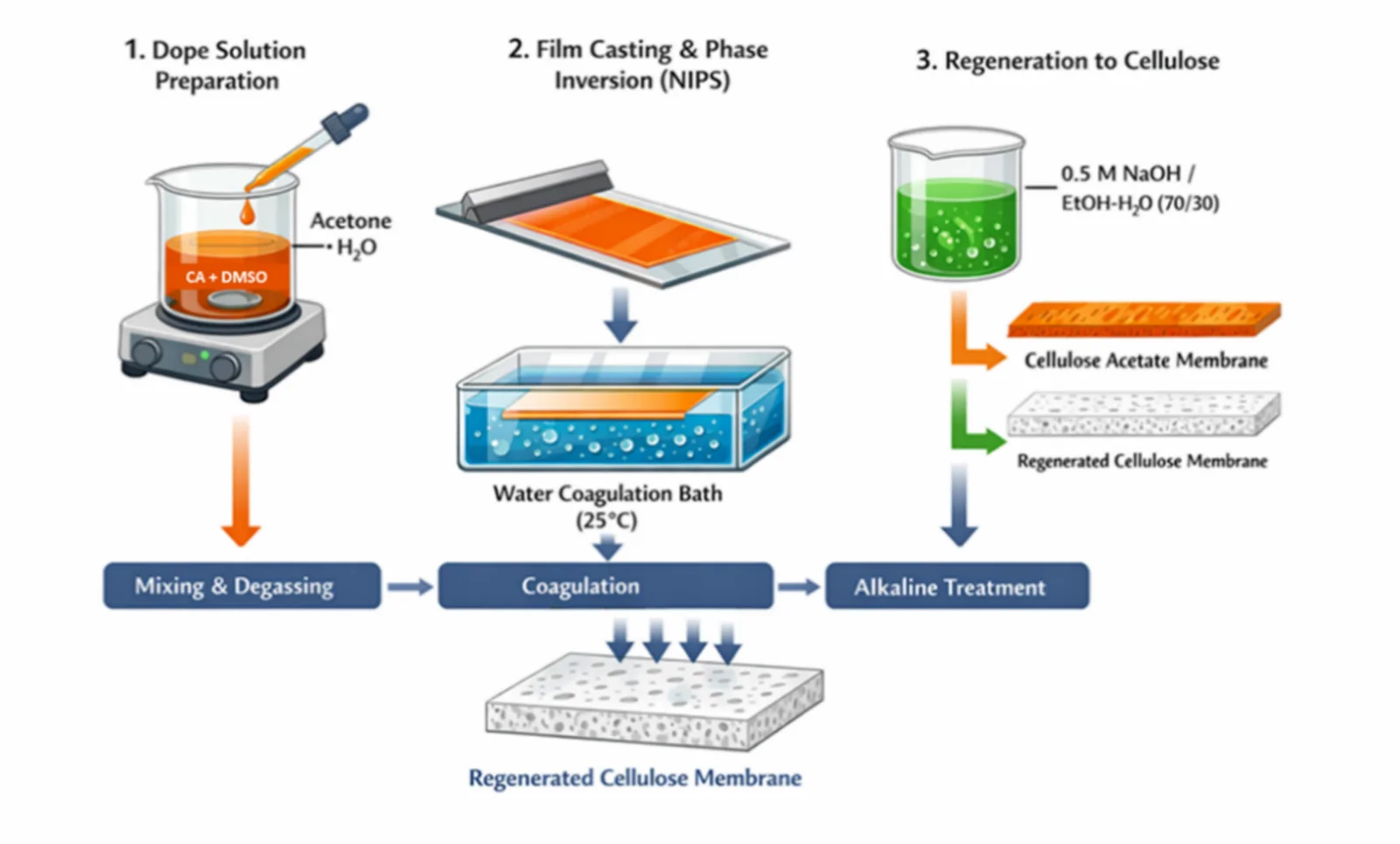
Schematic illustration of the fabrication of a monolithic regenerated cellulose membrane (RCM) with Janus-like properties for oil/water separation

Janus properties were imparted to regenerated cellulose monolithic membranes by selectively modifying one surface while preserving the intrinsic hydrophilicity of the opposite surface (Fig. 2). Before modification, hydrated membranes were gently blotted to remove excess surface water. The surface intended to remain hydrophilic was completely covered and protected with an ethanol-resistant PTFE tape layer, firmly applied and sealed along the edges to prevent any lateral penetration of the modifying solution.

The exposed surface was functionalized via a sol-gel silanization process as described in our previously reported work (Souare et al. 2024), using TEOS and MTES as precursors, with ethanol as the reaction solvent. The silanization sol was prepared by mixing 40 mL of ethanol with 5 mL of TEOS and 5 mL of MTES under magnetic stirring for 30 min. Subsequently, 5 mL of deionized water was added dropwise to initiate hydrolysis, followed by 1 mL of ammonium hydroxide (NH_4_OH) as a base catalyst. The solution was stirred for 12 h at room temperature, maintaining a pH of 9–11. The masked membrane was then brought into contact with the silanization solution on the exposed side, ensuring that the modification occurred predominantly on the exposed surface. TEOS acts as the silica network former, whereas MTES introduces methyl (-CH_3_) groups. Reducing surface energy and imparting hydrophobicity. After completion of the reaction, the membrane was removed, dried at ambient temperature, and the masking layer was carefully removed. The membrane was subsequently rinsed thoroughly with ethanol and deionized water to eliminate unreacted silane species, yielding a monolithic Janus-like membrane (Janus-like RCM) with a hydrophobic modified surface and a preserved hydrophilic opposite side, suitable for oil/water separations.

### Characterizations

#### Morphology and structure (SEM and EDS))

The surface morphology and elemental composition of the membranes were characterized using field-emission scanning electron microscopy (FE-SEM) coupled with energy-dispersive X-ray spectroscopy (EDS). The analyses were carried out using a Regulus 8100 FE-SEM system (Hitachi, Japan) equipped with a Bruker XFlash 6–30 silicon drift detector for EDS analysis. Before observation, membrane samples were cut into small sections and mounted onto aluminum stubs using conductive carbon tape. To improve electrical conductivity and prevent surface charging during imaging, the samples were coated with a thin conductive gold (Au) layer using a Hitachi MC1000 sputter coater. SEM images were recorded in secondary electron (SE) mode at an accelerating voltage of 3.0 kV and a working distance of approximately 5.1 mm, using magnifications of ×5,000, ×10,000, and ×20,000 corresponding to scale bars of 10, 5, and 2 μm, respectively. Elemental analysis was performed by EDS at an accelerating voltage of 20 kV and a working distance of approximately 15 mm. Both EDS spectra and elemental mapping were collected to determine the qualitative and semi-quantitative distribution of the main elements present on the membrane surface.

#### Surface chemical composition (FTIR)

Fourier transform infrared spectroscopy (FTIR) was employed to analyze the chemical structure and functional groups present on the membrane surface before and after modification. The spectra were recorded using a Nicolet 6700 FTIR spectrometer (Thermo Scientific, USA) equipped with an attenuated total reflection (ATR) accessory. Measurements were performed in the spectral range from 4000 to 400 cm^− 1^ with a resolution of 4 cm^− 1^, accumulating 32 scans for each sample to enhance the signal-to-noise ratio. All membranes were analyzed under identical conditions to ensure reliable comparison between the unmodified and silanized membranes.

#### Mechanical characterization

The tensile mechanical properties of the Janus-like monolithic regenerated cellulose membrane (Janus-like RCM) were evaluated to assess its structural robustness under deformation conditions relevant to oil/water separation applications. Samples (3 mm width, 0.5 mm thickness) were tested using an electronic tensile testing machine (Ningbo Weiheng Testing Instruments Co., Ltd., WH-5000, Ningbo, China) equipped with a 5 kN/100 N load transducer. The tensile test were performed using a gauge length of 31 mm at a crosshead speed of 5 mm/min. Before tensile testing, the specimens were pre-soaked in deionized water and tested in the water-wet state at room temperature to simulate the practical operating conditions of the membrane during oil/water separation. All experiments were performed in triplicate, and the reported values represent the mean of three independent measurements.

#### Surface wettability

The surface wettability of the membranes was evaluated by static water contact angle (WCA) measurements using a contact angle goniometer (JC2000D, Shanghai Zhongchen Powereach) at ambient temperature. A droplet of deionized water (typically 5 µL) was gently deposited onto the membrane surface, and the contact angle was recorded after stabilization for 10 s. Measurements were performed on both sides of the membrane to assess the asymmetric wettability induced by the unilateral surface modification. For each sample, contact angles were averaged over at least three independent measurements at different locations, and the values reported represent the average contact angle.

#### Nitrogen adsorption-desorption (BET) analysis

Nitrogen adsorption-desorption measurements were performed using a Micromeritics TriStar II Plus analyzer (version 3.03, USA). Prior to analysis, the samples were degassed at 40 °C for 12 h under vacuum (≤ 50 mTorr) to remove adsorbed contaminants. Isotherms were recorded at 77 K using nitrogen as the adsorbate over a range of relative pressures. The specific surface area was determined using the Brunauer-Emmett-Teller (BET) method. The pore size distribution and total pore volume were evaluated using the Barrett-Joyner-Halenda (BJH) method or density functional theory (DFT) models.

#### Oil /water separation capacity

A homemade oil/water separation setup was used to assess the performance of the Janus-like RCM membrane under gravity, without external pressure. The membrane, cut into a sample of 4.3 × 4.5 cm (effective area of 19.35 cm^2^), was fixed between two vertically aligned transparent tubes using a circular clamp. Before each separation, the membrane was fully wetted with deionized water, and the excess water was drained to condition the sample for testing. An oil/water mixture, consisting of 50 mL of water stained with methylene blue and organic phase stained with Sudan III, was gently poured into the upper tube until it came into contact with the membrane. The membrane was oriented with its hydrophobic side facing the feed and its hydrophilic side toward permeate, promoting selective water transport and retention of the organic phase. This configuration was used for both oils with densities lower than that of water and for denser solvents such as dichloromethane, which remain in direct contact with the membrane surface during separation. The process was followed from the initial contact of the mixture with the membrane until the complete cessation of flow. In all separation experiments, the permeate flux (F, Lm^−2^h^− 1^) and apparent volumetric oil-retention efficiency (η, %) were determined using Eqs. (1) and (2), respectively (Tian et al. 2023, Souare et al. 2024, Zhang et al. 2024).

1 $$Flux(L{m^{ - 2}}{h^{ - 1}})=\frac{V}{{St}}$$

2 $$\eta =\frac{{{V_{retained}}}}{{{V_{initial}}}} \times 100$$

where *V* is the volume of permeate collected (L), *S* is the effective membrane surface (m^2^), t is the separation time (hours), *V*_retained_ represents the volume of oil (organic phase) retained above the membrane after separation, and *V*_initial_ is the initial volume of the organic phase (oil) (L) introduced into the feed mixture.

#### Fouling resistance and reusability evaluation

The fouling resistance and the reusability of the Janus-like RCM membrane were evaluated through repeated gravity-driven oil/water separation cycles using water/hexane and water/dichloromethane mixtures (DCM). After each separation cycle, the membrane was rinsed with ethanol to remove residual oil from the membrane surface, followed by washing with deionized water. Before the next cycle, the membrane was prewetted again with deionized water. The flux recovery ratio (FRR) and the flux reduction ratio (FR) were calculated according to Eqs. (3) and (4), respectively (Li et al. 2026):

3 $$FRR(\% )=\frac{{{J_n}}}{{{J_0}}} \times 100$$

4 $$FR(\% )=\frac{{{J_0} - {J_n}}}{{{J_0}}} \times 100$$

where *J*_0_ is the initial flux of the membrane, and *J*_n_ is the recovered flux after the ^ℎ^ cycle of oil/water separation.

## Results and discussion

### Morphological and structural characterizations of monolithic Janus-like membranes

#### Morphological and structural characterization of monolithic Janus-like membranes

**Fig. 3:**
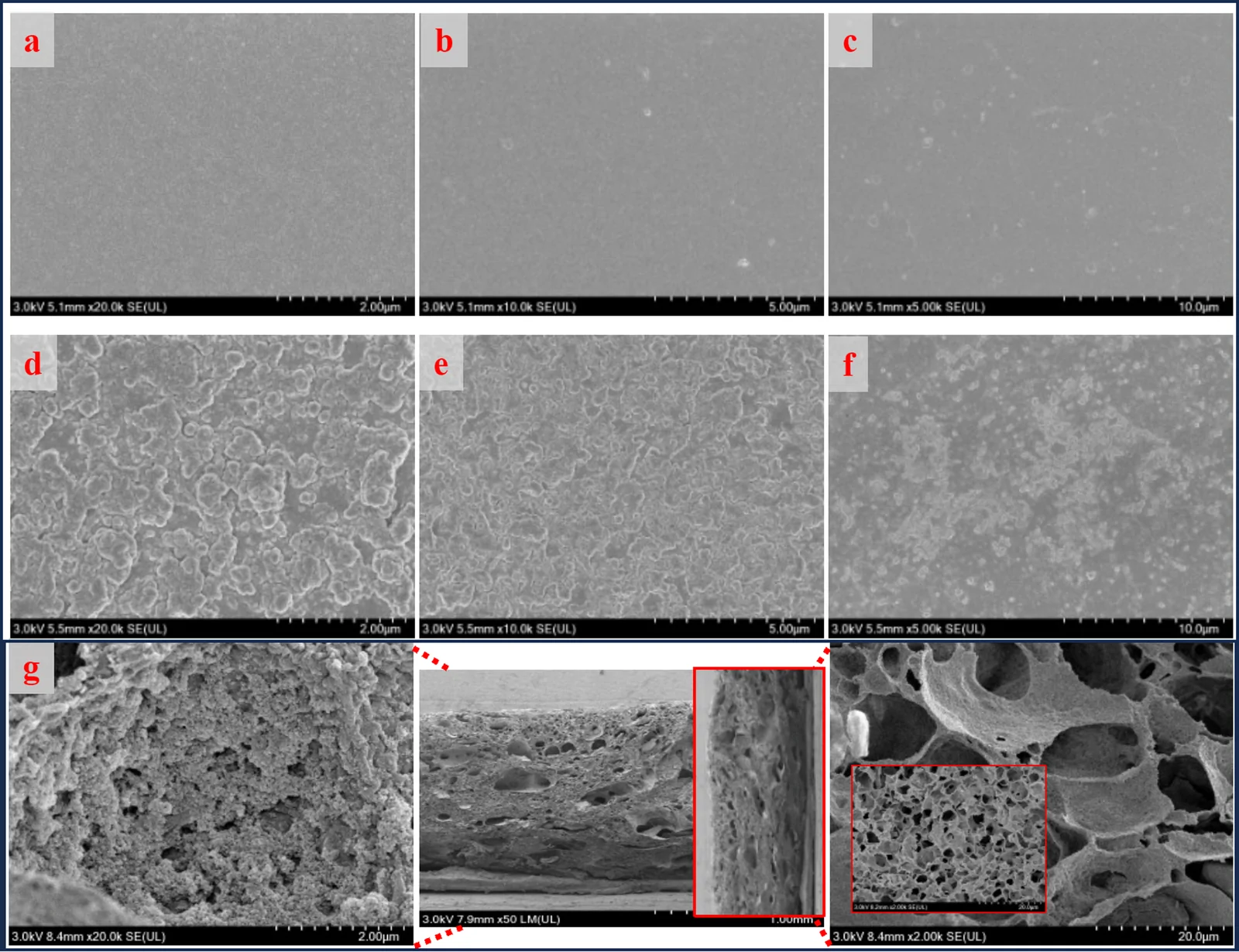
SEM of: RCM (**a-c**) and Janus-Like RCM (**d-f**), and (**g**) cross section of Janus-like RCM

The surface morphology and elemental composition of the membranes before (RCM) and after surface modification (Janus-like RCM) were studied using a field-emission scanning electron microscope (FE-SEM) coupled with energy-dispersive X-ray spectroscopy (EDS). SEM images of the RCM membrane are shown in Fig. 3a-c, corresponding to magnifications of x20,000, x10,000, and x5,000, respectively. In Fig. 3a, the membrane surface appears smooth, compact, and continuous, with no visible open pores at high magnification. Only minor surface irregularities are observed. In Fig. 3.b, the surface remains uniform and homogeneous over a wider observation area, with no sign of particulate deposits or aggregates. Similarly, Fig. 3.c shows a continuous defect-free morphology across a larger region of the membrane surface. The absence of visible pores at all magnifications suggests the formation of a dense, selective surface layer, which is typical of membranes fabricated via the non-solvent-induced phase separation (NIPS) process (Rahimpour et al. 2024). In such systems, the surface layer often contains nanometric pores that cannot be resolved under the present SEM imaging conditions.

In Fig. 3d, the membrane surface becomes rougher, with the appearance of granular and nodular features distributed across the surface. These structures become more apparent in Fig. 3e, where the membrane exhibits a heterogeneous surface texture over a broader area. In Fig. 3f, the modified membrane exhibits a textured and irregular morphology, characterized by numerous dispersed nodular structures (Wang et al. 2026). These morphological changes are attributed to the formation of a siloxane-based coating produced through the hydrolysis and condensation reactions of MTES and TEOS during the silanization process. The deposition of these silane-derived species increases surface roughness and partially covers the membrane surface. The cross-sectional SEM image (Fig. 3g, and S1) further reveals that the membrane exhibits a characteristic asymmetric structure. The region adjacent to the casting substrate appears relatively dense and displays a sponge-like morphology. Beneath this region, a porous substructure with interconnected macrovoids of various sizes is observed, which is typical of membranes prepared by the non-solvent-induced phase separation (NIPS) process (Wang et al. 2026). No apparent structural collapse or pore blockage was observed after the silanization treatment, suggesting surface modification preserves the internal porous structure of the membrane.

The AFM topographic image of the Janus-like RCM surface (supplementary file Figure S2) provides nanoscale information on the surface morphology after silanization. The modified membrane displays a three-dimensional topographical pattern consisting of peaks and valleys, with an average roughness (Ra) of 19.9 nm, root-mean-square roughness (Rq) of 25.1 nm, and maximum roughness height (Rmax) of 164 nm over a 1 × 1 µm^2^ scan area. The developed surface area was 25.7% higher than the projected area, indicating an enlarged effective interfacial area. This nanostructured morphology may enhance liquid–solid interactions and contribute to the asymmetric wettability of the Janus-like membrane.

**Fig. 4:**
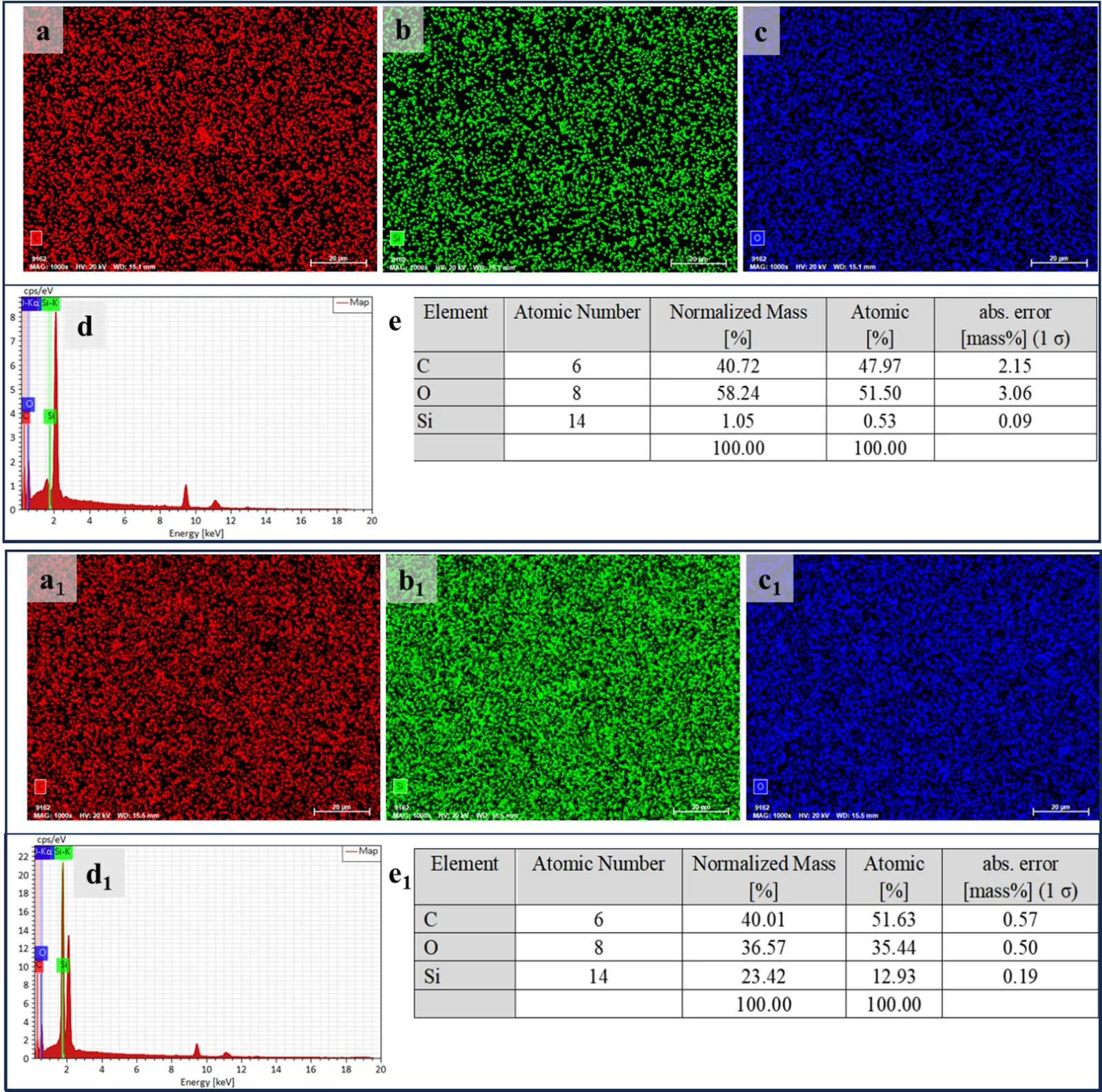
EDS elemental mapping and compositional analysis of the membranes. **a**-**c** Elemental distribution maps of carbon (red), oxygen (green), and silicon (blue) for the pristine RCM membrane; **d** corresponding EDS spectrum; **e** quantitative elemental composition; **a**_1_-**c**_1_ Elemental distribution maps of carbon (red), oxygen (green), and silicon (blue) for the Janus-like RCM membrane; **d**_1_ corresponding EDS spectrum; **e**_1_ quantitative elemental composition

To further verify the chemical composition of the membrane surfaces, EDS elemental mapping and spectral analysis were performed. The EDS results for the unmodified membrane are presented in Fig. 4a-e. The elemental mapping images show that carbon (C) and oxygen (O) are uniformly distributed throughout the analyzed region, which corresponds to the composition of regenerated cellulose. Only a weak silicon (Si) signal is detected, likely due to trace contamination introduced during the NIPS process, such as adsorption of silicate species from the coagulation bath or interaction with glassware. The increased surface reactivity of regenerated cellulose after deacetylation may further facilitate the adsorption of these trace elements. The corresponding EDS spectrum (Fig. 4d) shows dominant peaks for carbon and oxygen, while the quantitative analysis (Fig. 4e) indicates approximately 40.72 wt% carbon and 58.24 wt% oxygen, with only 1.05 wt% silicon, which is attributed to trace background signals.

For the modified membrane (Janus-like RCM), the EDS results are shown in Fig. 4a_1_-e_1_. The elemental mapping confirms that carbon and oxygen remain distributed across the membrane surface, indicating that the cellulose matrix is preserved after modification. However, a strong and uniformly distributed silicon signal is observed, demonstrating the successful incorporation of silicon-containing species onto the membrane surface. The EDS spectrum (Fig. 4d_1_) shows a silicon peak, while the quantitative analysis (Figure e_1_) indicates that the silicon content increases to approximately 23.42 wt%, accompanied by carbon and oxygen contents of 40.01 and 36.57 wt%, respectively.

To assess the silicon distribution within the Janus-like RCM membrane, cross-sectional SEM observation and EDS elemental mapping were performed (Figure S1a-k), together with a Si line scan (Figure S1l). The Si signal was higher near the silane-treated side and lower toward the protected side, which may indicate a non-uniform distribution across the membrane thickness. Surface EDS analyses were conducted on both membrane faces. The silane-treated surface (Figure S1m) showed a Si content of 23,42 wt%, whereas the protected surface (Figure S1n) showed a lower Si content of 6.13 wt%. The difference in Si concentration between the two surfaces indicates that silanization was mainly localized on the exposed surface, with limited migration of silane species toward the protected side.

Overall, the SEM, AFM, and EDS results show that the silanization treatment induces morphological and compositional changes on the silane-treated surface while preserving the structure of the regenerated cellulose membrane. The preferential localization of silicon on the treated side is consistent with the presence of siloxane-based coating derived from MTES and TEOS on the silane-treated surface.

#### FTIR characterization of functional groups

**Fig. 5:**
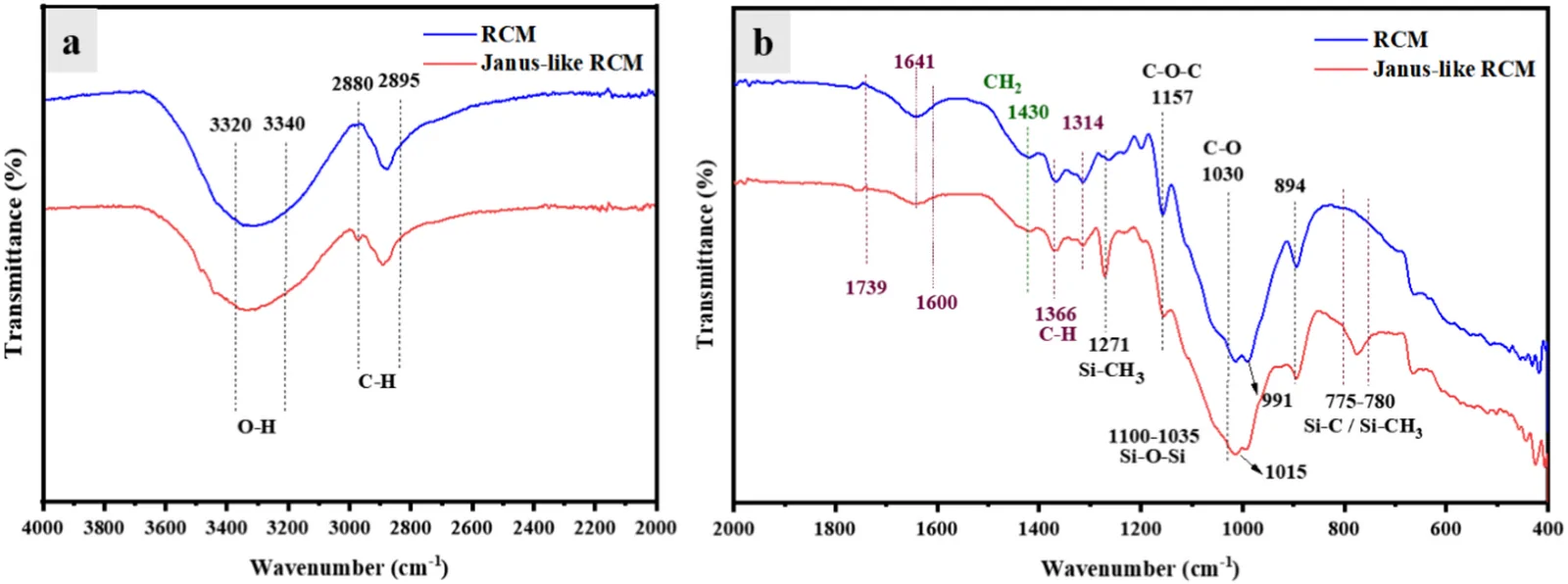
The FTIR spectra of RCM and Janus-like RCM in two spectral regions: **a** 4000 –2000 cm⁻¹ and **b** 2000 –400 cm⁻¹

FTIR analysis of the unmodified membrane (RCM) and the surface-modified Janus-like asymmetric wettability membrane (Janus-like RCM) are illustrated in Fig. 5. The FTIR spectra of the RCM and silanized Janus-like RCM membranes were analyzed in two spectral regions, namely 4000 –2000 cm^− 1^ and 2000 − 400 cm⁻¹, to better identify the chemical changes induced by surface modification. In the 4000 –2000 cm⁻¹ range (Fig. 5a), both membranes exhibited a broad absorption band centered around 3320–3340 cm⁻¹, which is attributed to the stretching vibration of hydroxyl groups from cellulose and absorbed water (Dudás et al. 2020). This band confirms the polysaccharide nature of the membrane matrix. The modified membrane showed a relative decrease in accessible hydroxyl contribution, suggesting partial coverage of the cellulose surface by the silane-derived layer. In addition, the band near 2880–2895 cm⁻¹, assigned to aliphatic C-H stretching, became more pronounced after modification, indicating the presence of methyl-containing organosilane groups introduced by MTES.

In the 2000 –400 cm⁻¹ region Fig. 5b, both membranes retained the characteristic cellulose bands at around 1430, 1366, 1157, and 1030 cm⁻¹, corresponding respectively to CH₂ bending, C–H deformation, glycosidic C–O–C stretching, and C–O stretching vibrations (Hospodarova et al. 2018, Cabrera et al. 2020, Taniyama and Takagi 2024). However, the modified membrane (Janus-like RCM) displayed additional or intensified bands around 1271, 1100 –1035 cm⁻¹, and 775–780 cm⁻¹. The band near 1271 cm⁻¹ can be assigned to Si–CH_3_ deformation (Dudás et al. 2020), while the strong contribution in the 1100 –1035 cm⁻¹ region is attributed to Si-O-Si asymmetric stretching, confirming the formation of a siloxane network through hydrolysis-condensation of TEOS and MTES (Almeida and Pantano 1990, Baldin et al. 2019). Furthermore, the more pronounced band at 775–780 cm^− 1^ is associated with Si-C and/or Si-CH₃ vibrations, providing additional evidence for the successful grafting of methylsilane species. The FTIR results demonstrate that the cellulose backbone remained preserved after treatment, while a siloxane-based hydrophobic layer was successfully introduced, predominantly on the silane-treated side of the membrane. These observations are in good agreement with wettability measurements, which showed that the modified surface exhibited a water contact angle of 91°, whereas the unmodified surface remained superhydrophilic with a WCA close to 0°.

### Textural and porous structure analysis

#### Adsorption-desorption isotherms and BET surface area analysis

**Fig. 6:**
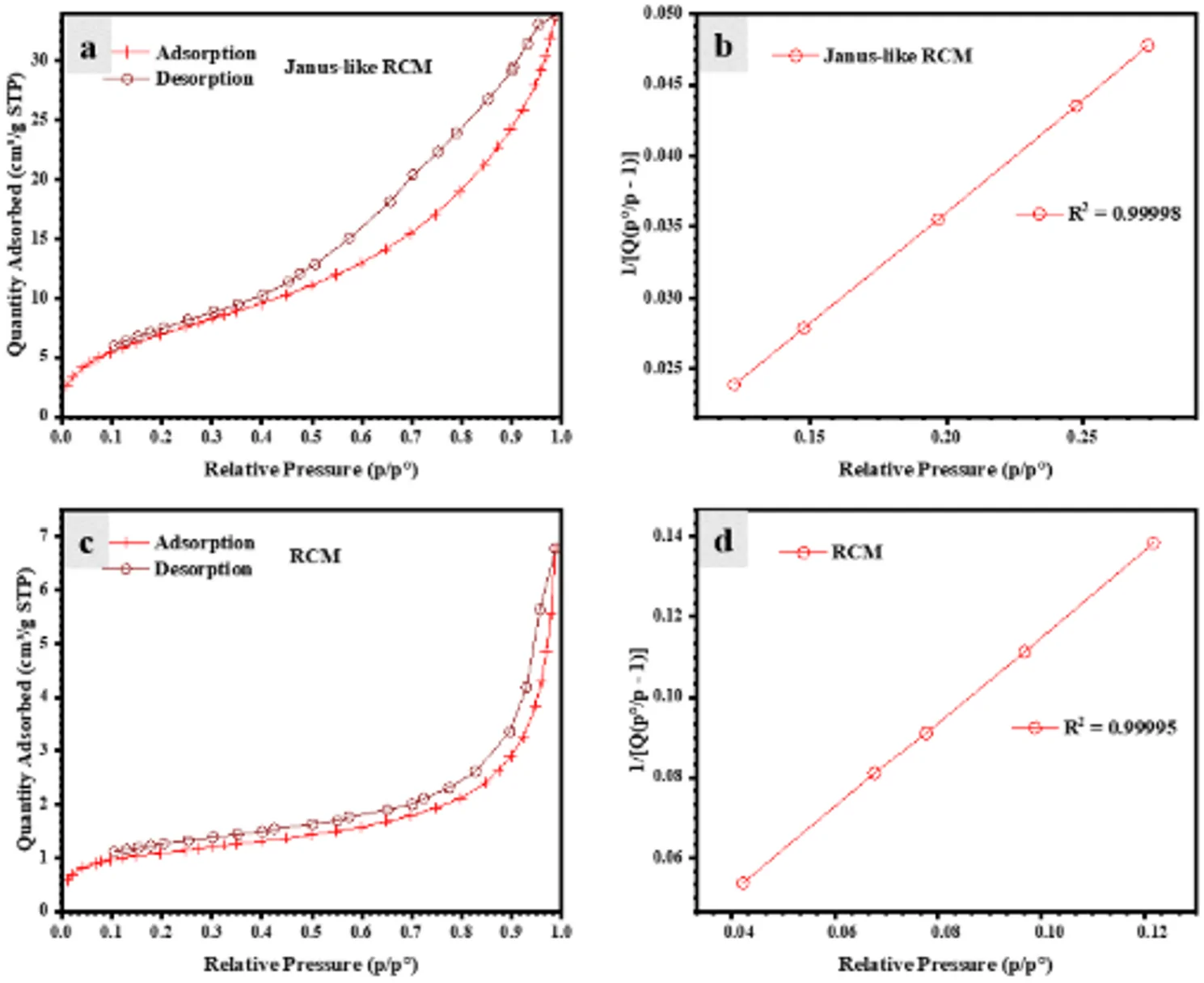
Nitrogen adsorption-desorption isotherms and corresponding BET surface area analysis of the membranes: **a** adsorption-desorption Isotherm and **b** linear BET plot of the Janus-like RCM membrane; **c** adsorption-desorption Isotherm and **d** linear BET plot of the pristine RCM membrane. All analyses were performed at 77 K

The nitrogen adsorption-desorption isotherms and the corresponding BET plots for the Janus-like RCM membrane are shown in Fig. 6a-b, with derived parameters summarized in Table 1. (More detailed data, including the analysis of the isotherms and the derived BET and BJH parameters, are provided in Table S1 (supplementary file)). The Janus-like RCM membrane shows an improved adsorption capacity across the entire pressure range, reaching 33.85 cm^3^g^− 1^ STP at P/P₀ ≈ 0.99. The observed hysteresis loop suggests capillary condensation in nitrogen-accessible mesoporous domains or voids associated with the modified surface layer (Sing 1985, Lowell et al. 2004). This enhanced adsorption behavior may be associated with the MTES/TEOS treatment, which introduced siloxane groups (Si-O-Si), as confirmed by FTIR analysis. The BET analysis (Fig. 6d) exhibits a six-fold increase in specific surface area (26.92 m^2^g^− 1^), and a higher monolayer capacity (*Q*_*m*_ = 6.18 cm^3^g^− 1^ STP) compared to the pristine (RCM). As the silanization treatment was predominantly localized on the exposed surface of the membrane, the increase in nitrogen uptake and BET surface area is likely associated with changes in the nitrogen-accessible properties of the siloxane-containing surface layer, including its morphology and adsorption-site accessibility, rather than with changes throughout the cellulose matrix.

For reference, the pristine RCM membrane exhibits a lower adsorption capacity (maximum 6.77 cm^3^g^− 1^ STP at *P/P*_*0*_ ≈ 0.99) a nd a lower specific surface area (*S*_*BET*_ = 4.06 m²g^− 1^), reflecting limited nitrogen-accessible adsorption sites (Fig. 6c-d) (Rouquerol et al. 2013, Mir et al. 2022). Overall, the Janus modification alters surface chemistry, nitrogen adsorption behavior, and BET surface area of the membrane, indicating changes in the nitrogen-accessible properties of the modified surface.

**Table 1:** Textural properties of the pristine RCM and modified Janus-like RCM membranes

Samples	S_BET_ (m^2^/g)^a^	BET constant (C)	Vpore (cm^3^/g)^b^	Dpore (nm)^c^	*R* ^2^
RCM	4.06	122.2	0.0098	15.56	0.99995
Janus-like RCM	26.92	34.6	0.0535	7.22	0.99998

^a^BET specific surface area. ^b^BJH cumulative adsorption pore volume. ^c^Average BJH adsorption pore diameter.

#### BJH structure analysis: distribution and cumulative volume

**Fig. 7:**
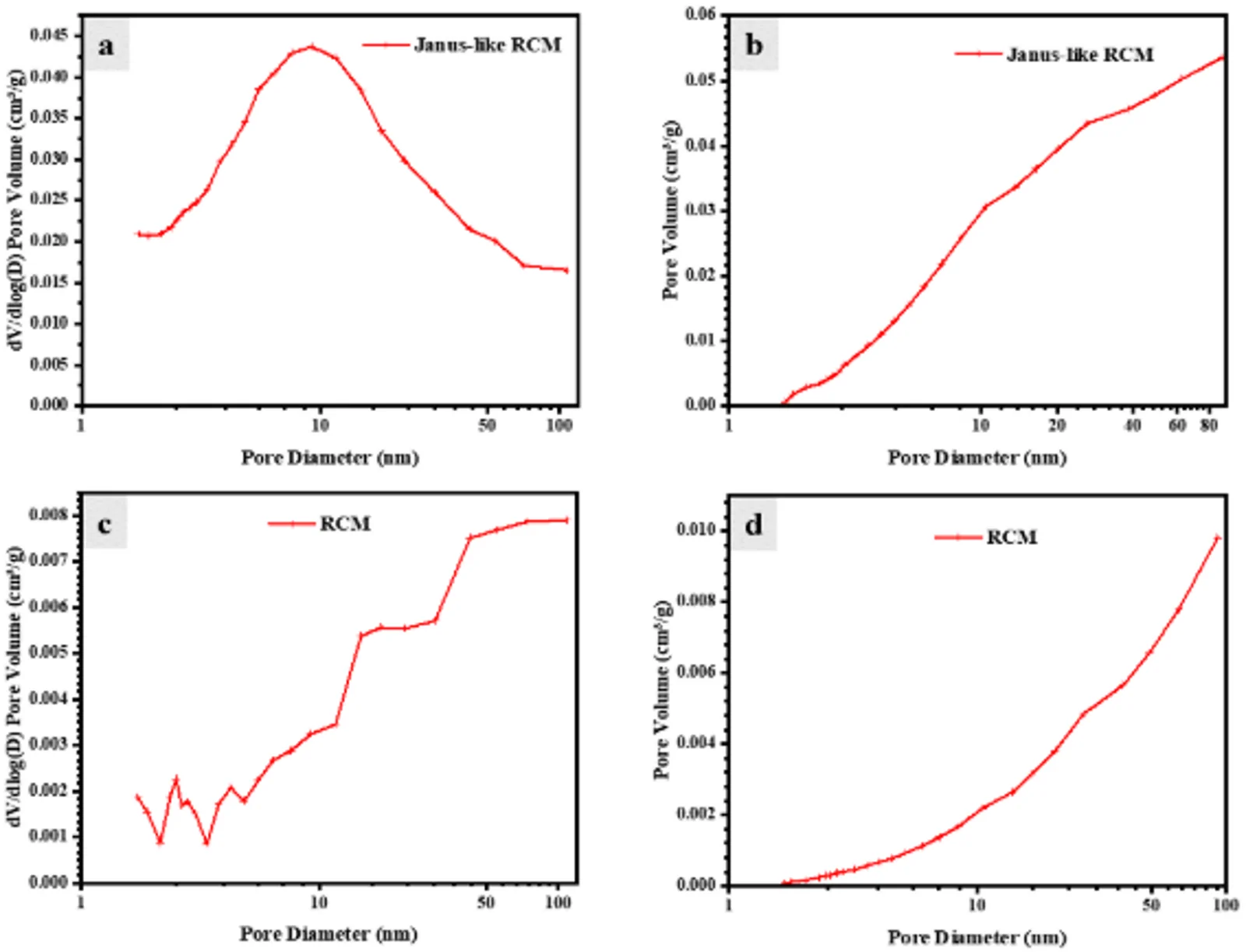
BJH pore structure analysis of the membrane. **a** Pore size distribution (dV/dlog(D)) and **b** cumulative pore volume of the Janus-like RCM membrane; **c** pore size distribution and **d** cumulative pore volume of the pristine RCM membrane

The pore size distribution and cumulative pore volume of membranes, determined using the Barrett-Joyner-Halenda (BJH) method, are presented in Fig. 7.

The Janus-like RCM membrane (Fig. 7a-b) exhibits a shift in pore structure compared to RCM. The main distribution peak is shifted toward the nanoscale (9.15 nm), with higher pore-volume contributions (dV/dlog(D)) for pores between 5.50 and 7.54 nm, indicating a greater contribution of mesopores within the 5–10 nm range. This change in the pore network suggests that the MTES/TEOS surface functionalization contributes to the observed mesoporosity and pore accessibility. The increase in pore volume may be related to changes in surface morphology, pore accessibility, and the organization of the deposited layer, in addition to the presence of siloxane species. Cumulative pore volume analysis shows this trend, with a five-fold increase for Janus-like RCM (0.0535 cm^3^g^− 1^) relative to RCM (0.00982 cm^3^g^− 1^; Fig. 7c-d). This higher cumulative pore volume across the pore-diameter range may reflect changes in pore accessibility and the organization of the deposited layer. Overall, these results indicate that the Janus modification alters the accessible membrane porosity toward the nanometric scale, which may contribute to water/oil separation performance.

### Tensile properties

**Fig. 8:**
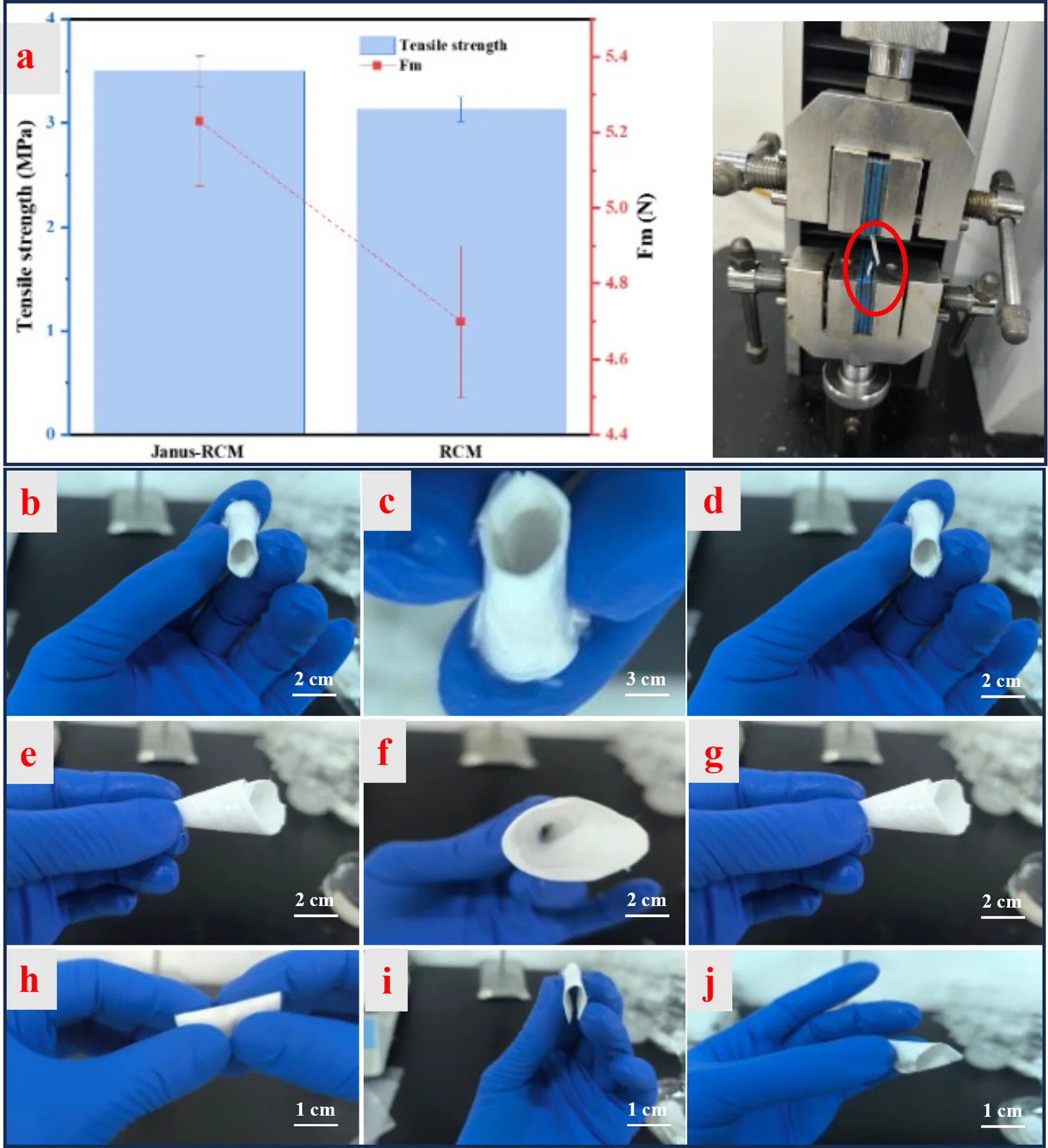
Mechanical flexibility of the Janus-like RCM membrane under different deformation modes: **a** Tensile strength, **b-d** axial rolling, **e-g** conical deformation, **h** and **i** bending, and **j** symmetric bidirectional bending from diametrically opposed edges

Mechanical tests were performed to evaluate the mechanical properties of the prepared membranes, and the corresponding results are presented in Fig. 8a. The tensile strength (TS) was calculated according to TS = Fₘ / S₀, where Fₘ is the maximum breaking force, and S₀ is the initial cross-sectional area of the specimen. The RCM membrane exhibited a tensile strength of 3.13 MPa with a maximum breaking force of 4.70 N, whereas the Janus-like RCM membrane exhibited slightly higher values of 3.50 MPa and 5.23 N, respectively. This moderate improvement is likely associated with the formation of siloxane groups on the modified surface. These groups may contribute to interfacial interactions and stress transfer during tensile loading while maintaining the monolithic porous structure of the regenerated cellulose membrane. These results are comparable to those reported in the literature (Lin et al. 2025, Ramírez-Colón et al. 2022, Zhang et al. 2022). Furthermore, the mechanical tolerance during handling, installation, and operation was evaluated through various deformation modes, including axial rolling, conical deformation, bending, and bidirectional bending (Fig. 8a-i; supplementary videos T-S1 and T-S2).

Under all deformation modes, the membrane exhibited no visible cracking, delamination, or macroscopic failure. Axial rolling revealed homogeneous flexibility, while localized and multi-directional deformations did not compromise material cohesion or interfacial adhesion between the silanized layer and the regenerated cellulose support. The preservation of structural integrity under combined tensile, compressive, and shear stresses confirms that unilateral surface modification does not adversely affect the membrane’s overall mechanical stability, ensuring reliable performance during oil/water separation processes.

### Asymmetric wettability and practical separation performance of the Janus-like RCM membrane

**Fig. 9:**
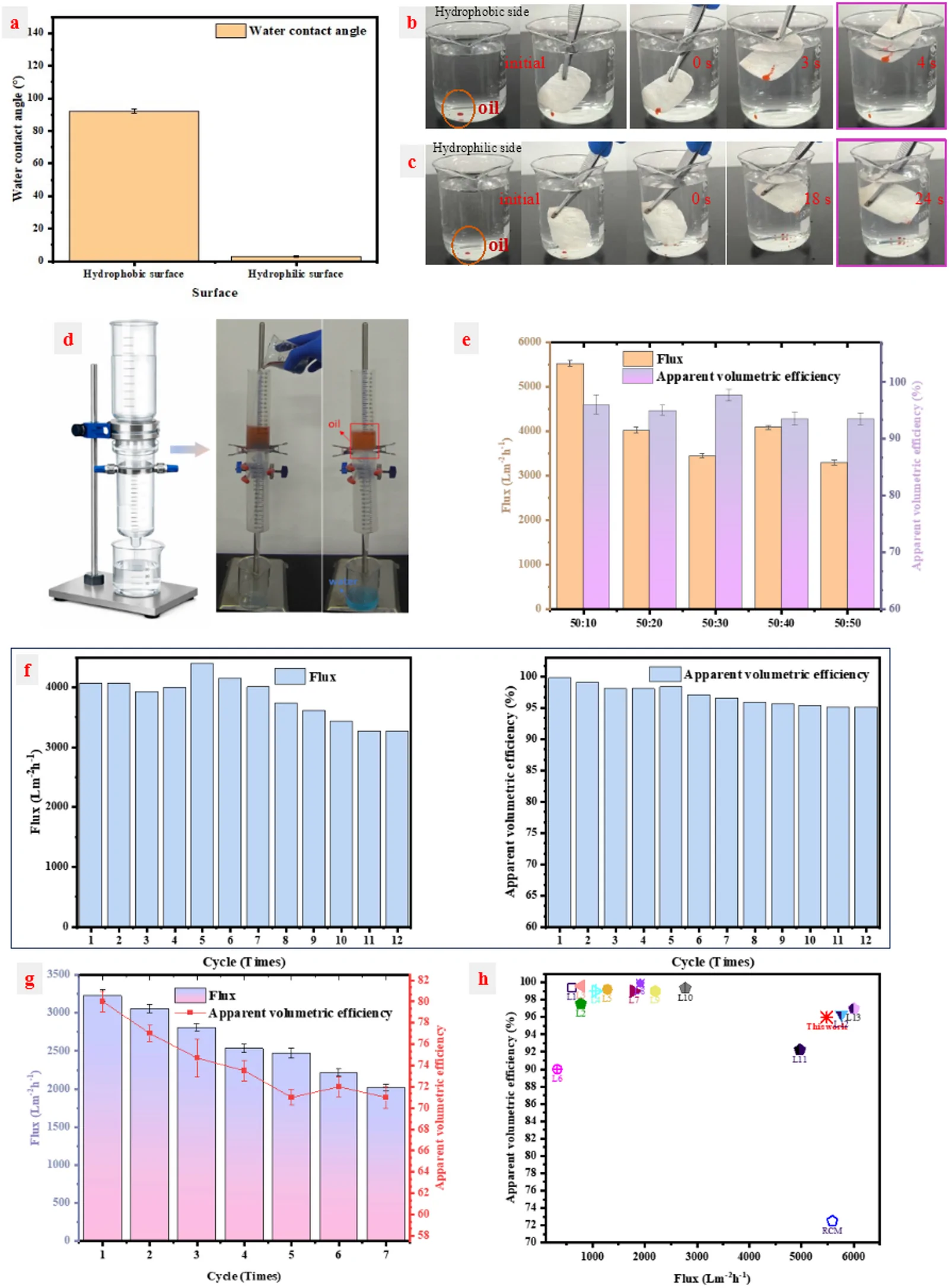
Surface wettability characteristics and gravity-driven oil/water separation performance of the as-prepared Janus-like RCM membranes: **a** Water contact angle (WCA) on the hydrophobic (silanized) and hydrophilic (unmodified) sides. **b** Underwater oil capture behavior of the hydrophobic side and **c** oil rejection behavior of the hydrophilic side. **d** Oil-water separation device. **e** Permeation flux and apparent volumetric oil-retention efficiency as a function of oil volume (10–50 mL) in the aqueous phase for the water/peanut oil system. **f** Permeate flux and apparent volumetric oil-retention efficiency during 12 consecutive water/hexane separation cycles. **g** Evaluation of flux and apparent volumetric oil-retention efficiency for the water/dichloromethane (DCM) mixture. **h** Comparative analysis of the Janus-like RCM separation performance with various Janus membranes reported in the literature

The oil/water separation capability of the Janus-like RCM membrane is primarily governed by its asymmetric surface wettability. As shown in Fig. 9a, the membrane exhibits a clear wettability contrast between its two faces. The modified side displays a water contact angle (WCA) of 91°, indicating a hydrophobic character resulting from the grafting of a low surface energy silane group (Ji et al. 2017). In contrast, the opposite side retains its intrinsic hydrophilicity, with instantaneous water spreading (WCA ≈ 0°). This wettability gradient is further enhanced by the membrane microstructure, which, according to the Wenzel model, amplifies the intrinsic surface properties (Deng et al. 2026, Li et al. 2023, Xiao et al. 2025). The combination of the surface chemistry and microstructural features confirms the successful fabrication of an asymmetric Janus-like monolithic with distinct wetting characteristics.

When the pre-wetted hydrophobic side contacts an oil droplet (dyed with Sudan III) underwater, it rapidly absorbs the oil while effectively repelling the aqueous phase (Figs. 9b, Supplementary Film S-H). Consequently, the hydrophilic side promotes water permeation and effectively rejects oils (Figs. 9c, Supplementary Film S-h). This bifacial functionality enables efficient separation, as demonstrated by the membrane’s capacity to block 50 mL of oil while allowing the rapid permeation of 50 mL of methylene blue-dyed water (Supplementary Film S5).

The oil/water separation test was carried out using a gravity-driven filtration system without external pressure (Fig. 9d). For the water/peanut oil system, five consecutive tests were conducted by incrementally increasing the oil volume from 10 to 50 mL while maintaining a constant aqueous phase of 50 mL. The Janus-like RCM membrane achieved an apparent volumetric oil-retention efficiency up to 95%, with permeation fluxes ranging from 3300.83 to 5526.68 Lm^−2^h^− 1^ (Fig. 9e). Although a slight flux decline was observed with increasing oil volumes, likely due to interfacial resistance and partial pore clogging, the apparent volumetric oil-retention efficiency remained consistently high, indicating effective oil retention. For the water/hexane (10 mL oil:50 mL water), the membrane exhibited an average permeation flux of 3950.2 Lm^−2^h^− 1^ with an apparent volumetric oil-retention efficiency of 96.93% over 12 consecutive cycles (Fig. 9f). In contrast, over seven consecutive separation cycles using a water/dichloromethane (DCM) mixture (50 mL water:10 mL DCM), the system exhibited more moderate performance (Fig. 9g), with an average flux of 2620.78 Lm^−2^h^− 1^ and an apparent volumetric oil-retention efficiency of 70.14%. This difference in performance is attributed to the high density and low viscosity of DCM, which facilitates partial breakthrough under hydrostatic pressure. As reference, the unmodified RCM could also separate oil/water with a high flux of 5590 Lm^−2^h^− 1^ but showed a lower apparent volumetric oil-retention efficiency of 72.5% compared to the Janus-like RCM, which shows the impact of functionalization on the Janus-like RCM. A comparison with the Janus membranes described earlier (Fig. 9h, Table S2) shows that the Janus-like RCM membrane offers comparable performance, although it operates solely by gravity (Zhang et al. 2024, Hou et al. 2025, Fei et al. 2024, Cheng et al. 2023, Zhu et al. 2024). Overall, the membrane demonstrates high apparent volumetric oil-retention efficiency for water/peanut oil and water/hexane systems, whereas a lower value was observed for the water/DCM system, indicating that membrane performance depends on the physicochemical properties of the organic phase (density and viscosity).

When a water droplet contacts the silanized surface, the apparent contact angle slightly exceeds 90°, indicating a hydrophobic wetting state where cos*θ*_*h*_ < 0. According to the Young-Laplace equation, P_c_= (2γ cos*θ*)/r, where *P*_c_ is the capillary pressure, γ is the interfacial tension, θ is the contact angle, and r is the effective pore radius (Su et al. 2025). This indicates that spontaneous water intrusion is not favored on the hydrophobic surface, and a capillary entry barrier must be overcome at the pore entrance (Li et al. 2025, Sh et al. 2023, Yang et al. 2018, Ghalavand et al. 2025). The Janus-like RCM membrane exhibits a refined mesoporous architecture, with an increased specific surface area (26.92 vs. 4.06 m^2^ g^− 1^), increased pore volume (0.0535 vs. 0.0098 cm³ g⁻¹), and a reduced average pore diameter from 15.6 nm (RCM) to 7.2 nm (Janus-like RCM). This reduction in pore radius may increase the magnitude of capillary effects according to the Young-Laplace relationship, thereby enhancing capillary effects within the interconnected pore network (Lin et al. 2025).

Before separation, the membrane is prewetted, ensuring that the mesoporous channels are saturated with water. In this state, transport is influenced by capillary pressure asymmetry across the Janus-like structure rather than initial pore infiltration. The hydrophilic permeate side (WCA ≈ 0°, cos θ ≈ 1) favors water imbibition through capillary suction, whereas the hydrophobic feed-facing side provides a capillary entry barrier against spontaneous water intrusion. This wettability contrast may generate an asymmetric capillary pressure difference across the membrane (Zheng et al. 2022):

5 $$ \Delta {\mathrm{P}}_{{\mathrm{c}}} = {\mathrm{P}}_{{\mathrm{c}}} ,_{{{\mathrm{hydrophilic}}}} - {\text{ P}}_{{\mathrm{c}}} ,_{{{\mathrm{hydrophobic}}}} $$

which could facilitate directional water transport through the interconnected mesoporous pathways (Tian et al. 2023, Meng et al. 2023). Meanwhile, oil transport is expected to be restricted by the water-filled pore network and capillary resistance within the nanoscale pores. Under these wet conditions, the hydrophobic surface may contribute to interfacial resistance during liquid displacement, suggesting a qualitative mechanism based on the observed wettability contrast and pore structure. Thus, the separation performance of the Janus-like RCM membrane arises from the combination of asymmetric wettability and mesoporous structure, enabling capillary-assisted directional water transport while limiting oil permeation under gravity-driven conditions.

### Fouling resistance and reusability

The fouling resistance and reusability of the Janus-like RCM membrane were evaluated through repeated gravity-driven oil/water separation cycles followed by a simple cleaning step. After each separation cycle, the membrane was rinsed with ethanol to remove residual oil droplets, washed with deionized water, and then prewetted again before reuse (Tian et al. 2023, Ma et al. 2019).

For the water/hexane mixture, the membrane maintained stable performance over 12 cycles. The recovered flux decreased from 4070.00 to 3265.31 Lm^−2^h^− 1^, corresponding to a flux recovery ratio (F_RR_) of 80.23% and a flux reduction ratio of 19.77%. Meanwhile, the apparent volumetric oil-retention efficiency remained above 95%, confirming a certain fouling resistance and reusability. For the water/DCM mixture, the recovered flux decreased from 3224.81 to 2016.74 Lm^−2^h^− 1^ after 7 cycles, corresponding to an F_RR_ of 62.54% and a flux reduction ratio of 37.46%. The lower recovery observed for DCM is likely related to its higher density and greater tendency to penetrate or accumulate within the membrane pores under hydrostatic pressure. In addition, the peanut oil/water tests confirmed the membrane tolerance toward increasing oil loading. When the peanut oil volume increased from 10 to 50 mL while maintaining 50 mL of water, the apparent volumetric oil-retention efficiency remained above 93.5%, indicating a certain resistance to oil accumulation and pore blockage. These results indicate that the Janus-like RCM membrane exhibits oil-phase-dependent fouling behavior and oil-loading (peanut oil) tolerance under gravity-driven oil/water separation conditions.

## Conclusion

In this work, a monolithic Janus-like membrane was developed from cellulose acetate using a rational design strategy that decouples the porous structure from the surface chemistry. The combination of the NIPS process, alkaline deacetylation, and silanization predominantly localized on the exposed surface using TEOS/MTES produced a Janus-like RCM membrane with a continuous architecture and asymmetric wettability, without relying on conventional multilayer structures. Morphological, chemical, and textural analyses (SEM, EDS, FTIR, BET/BJH) confirmed the presence of a siloxane-containing surface layer predominantly localized on the exposed surface, the redistribution of pores toward the nanoscale, and an increase in specific surface area. The resulting Janus-like RCM showed a tensile strength of 3.50 MPa and a maximum breaking force of 5.23 N, comparable to or even superior to some Janus membranes reported in the literature. For oil/water separation, the membrane exhibited separation fluxes ranging from 3300.83 to 5527.68 Lm^−2^h^− 1^, with an apparent volumetric oil-retention efficiency of 95% for water/peanut oil, while maintaining good mechanical properties. For the water/hexane mixtures, the membrane achieved an average separation flux of 3950.62 Lm^−2^h^− 1^ with an apparent volumetric oil-retention efficiency of 96.93%. In contrast, for the water/DCM system, the separation flux remained moderate at 2620.78 L m^− 2^ h^− 1^ with an average apparent volumetric oil-retention efficiency of 70.14%. The directional transport mechanism, governed by a capillary pressure gradient induced by asymmetric wettability, ensured selective and spontaneous permeation of water under simple gravity, without external energy input. Due to its simplified manufacturing process, this membrane offers an alternative to conventional Janus membranes.

## Supplementary Information

Below is the link to the electronic supplementary material.

Supplementary Material 1

Supplementary Material 2

Supplementary Material 3

## Data Availability

The data that support the findings of this study are available from the corresponding author upon reasonable request.
