# Supplementary material for: Design of a monolithic cellulose Janus-like membrane for oil/water separation
Source: Bioresour Bioprocess. 2026 Oct 9;13(1):148. doi: 10.1186/s40643-026-01142-0 (PMC13653026; doi:10.1186/s40643-026-01142-0)
Supplement: Supplementary file 1 [file 40643_2026_1142_MOESM1_ESM.docx]

**Design of a Monolithic Cellulose Janus-like Membrane for oil/water separation**

Mamadou Souare^1*^, Changqing Dong^1,2*^, Xiaoying Hu^1^, Junjie Xue ^1^, Junjiao Zhang^3^,

1 National Engineering Laboratory for Biomass Power Generation Equipment, School of New Energy, North China Electric Power University, Beijing 102206, China
2 State Key Laboratory of Alternate Electrical Power System with Renewable Energy Sources, North China Electric Power University, Beijing 102206, China
3 School of Energy, Power and Mechanical Engineering, North China Electric Power University, Beijing 102206, China

The SEM-EDS analysis performed on both surfaces of the Janus-like monolithic membrane revealed an asymmetric distribution of silicon. The silane-treated surface exhibited a higher Si content (23.42 wt%), supporting the incorporation of MTES/TEOS-derived siloxane species (Fig. S1 m). In comparison, the protected surface showed a lower Si content (6.13 wt%), indicating limited silane diffusion or migration during the modification process (Fig. S1 n). The difference in Si concentration between the two surfaces indicates that silanization mainly occurred on the exposed surface rather than uniformly throughout the membrane. This asymmetric elemental distribution is consistent with the different wettability characteristics of the two sides, where the modified surface exhibited a water contact angle of approximately 91°, while the protected surface remained hydrophilic with a near-zero water contact angle. These results support the fabrication of a Janus-like monolithic membrane with asymmetric surface modification.

Figure S1. Cross-sectional SEM-EDS characterization and surface elemental analysis of the Janus-like RCM membrane: (a-f) SEM images of the membrane cross-section; (g-k) elemental maps of C, O, and Si; (l) EDS line scan showing the Si intensity profile across the cross-section of the membrane; (m) SEM-EDS analysis of the silane-treated surface; (n) SEM-EDS analysis of the protected surface. The comparative surface analysis reveals an asymmetric distribution of silicon between the two faces of the membrane, with a higher Si content detected on the silane-treated surface.

Figure S2. AFM height images of the Janus-like RCM membrane: (a) 2D topography and (b) 3D topography (scan area: 1×1 µm²).

Table S1 Comparative summary of BET and BJH textural properties of RCM and Janus-RCM membranes

| **Parameter** | **RCM** | **Janus-like RCM** |
| --- | --- | --- |
| **Adsorbate** | N₂ | N₂ |
| **Analysis Bath Temperature** | 77.350 K | 77.350 K |
| **Sample Mass** | 0.1709 g | 0.1909 g |
| **BET Surface Area** | 4.0630 m²/g | 26.9179 m²/g |
| **BET Surface Area (Single Point)** | 3.8294 m²/g | 24.9658 m²/g |
| **Single Point p/p°** | 0.121713812 | 0.273985182 |
| **Langmuir Surface Area** | 4.5637 m²/g | 26.9244 m²/g |
| **Qm (BET)** | 0.9335 cm³/g STP | 6.1844 cm³/g STP |
| **BET Constant (C)** | 122.215966 | 34.619429 |
| **BET Correlation Coefficient** | 0.9999497 | 0.9999826 |
| **Micropore Volume (t-plot)** | 0.000566 cm³/g | 0.001922 cm³/g |
| **Micropore Surface Area (t-plot)** | 1.4774 m²/g | *Not reported* |
| **External Surface Area (t-plot)** | 2.5857 m²/g | 26.9484 m²/g |
| **Thickness Equation** | Harkins and Jura | Harkins and Jura |
| **Total Pore Volume (Single Point Adsorption)** | 0.010482 cm³/g | 0.052364 cm³/g |
| **Single Point Total Volume Condition** | < 156.7635 nm at p/p° = 0.987619380 | < 168.2894 nm at p/p° = 0.988480445 |
| **BJH Cumulative Pore Volume (Adsorption)** | 0.009823 cm³/g | 0.053531 cm³/g |
| **BJH Adsorption Range** | 1.5000 to 297.7657 nm | 1.5000 to 297.7657 nm |
| **BJH Cumulative Pore Volume (Desorption)** | 0.010247 cm³/g | 0.053245 cm³/g |
| **BJH Desorption Range** | 1.0000 to 300.0000 nm | 1.0000 to 300.0000 nm |
| **BJH Cumulative Surface Area (Adsorption)** | 2.5254 m²/g | 29.6713 m²/g |
| **BJH Cumulative Surface Area (Desorption)** | 3.6136 m²/g | 37.8271 m²/g |
| **Average Pore Diameter (4V/A, BET) - Adsorption** | 10.3190 nm | 7.7812 nm |
| **Average Pore Diameter (4V/A, BET) - Desorption** | 10.3190 nm | 7.7812 nm |
| **Average Pore Diameter (BJH)-Adsorption** | 15.5595 nm | 7.2166 nm |
| **Average Pore Diameter (BJH) - Desorption** | 11.3421 nm | 5.6304 nm |

Table S 2	Comparison of the developed Janus-like membrane with Janus membranes reported in the literature

| **Reference** | **Membrane type** | **Fabrication method** | **Feed type** | **Driving force** | **Membrane dimension** | **Flux (L m⁻² h⁻¹)** | **Definition of separation efficiency** | **Efficiency (%)** |
| --- | --- | --- | --- | --- | --- | --- | --- | --- |
| L1[1] | Stainless steel mesh Janus membrane (ZIF-8 based) | Electrodeposition + in-situ growth + surface modification | Oil/water emulsions (W/O and O/W) | Gravity-driven | NR | 601 (W/O); 62 (O/W) | NR | 99.1 (W/O); 99.7 (O/W) |
| L2[2] | SBS Janus membrane | Film lamination strategy (hydrophobic/hydrophilic layers) | Oil/water mixture and oil/water emulsion | Gravity-driven | Effective area: 10 cm²; thickness: 290 μm | 870.2 (mixture); 680 (emulsion) | Water recovery efficiency based on collected water mass | 99.91 (mixture); 95.23 (emulsion) |
| L3[3] | Wood Janus membrane | Biomimetic design | Oil/water emulsions (W/O and O/W) | Vacuum-assisted (0.095 MPa) | NR | 810 (W/O); 747 (O/W) | Concentration-based rejection efficiency | >99.6 |
| L4[4] | PVDF-based Janus membrane (TA-PEI/TA-Cu²⁺ coating) | Double-cross-linked polyphenol hydrophilic layer coating | Surfactant-stabilized oil/water emulsions (O/W and W/O) | Vacuum-assisted (O/W: 0.09 MPa); pressure-driven (W/O: 0.01 MPa) | NR | 1061 | Concentration-based rejection efficiency | >99 |
| L5[5] | Bioinspired fibrous Murray membrane (core-shell fibers) | Eccentric electrospinning | Oil/water emulsions | Gravity-driven (10 cm liquid head) | NR | >1282 | Concentration-based oil rejection efficiency | >99.2 |
| L6[6] | PLA nanofiber Janus membrane | Electrospinning | Water/CCl₄ mixture; petroleum ether-in-water emulsion; water-in-petroleum ether emulsion | Gravity-driven | NR | 21073.57 (water/CCl₄); 1792.81 (O/W); 974.36 (W/O) | Concentration-based separation efficiency | 97.5; 97.04; 95.31 |
| L7[7] | cPVA-PVDF/PMMA/GO Janus membrane | Layer-by-layer electrospinning | Water-in-oil emulsions (n-hexane/water and diesel/water) | Gravity-driven | NR | 1909.9 | Gravimetric oil recovery efficiency | 99.9 |
| L8[8] | Integrated Janus membrane (MHCH/SSM-Co₃O₄) | Assembly method | Surfactant-stabilized oil/water emulsions (O/W and W/O) | Vacuum-assisted (W/O: 0.06 MPa; O/W: 0.08 MPa) | Effective area: 9.62 cm²; thickness: ~350 μm | 1100–3300 | Concentration-based rejection efficiency | >99 |
| L9[9] | Biodegradable Janus membrane | Electrospinning + plasma treatment | Oil/water mixtures | Gravity-driven | NR | 2779.9 | Gravimetric oil recovery efficiency | 99.28 |
| L10[10] | Polyester fabric (D4H-PF) | Plasma-induced polymerization | n-Hexane/water and DCM/water mixtures | Gravity-driven | Effective area: 10 cm² | 4968.15 (n-hexane/water); 4872.61 (DCM/water) | Water recovery efficiency based on collected water mass | 98.88 (n-hexane/water); 92.24 (DCM/water) |
| L11[11] | Metal-organic framework (MOF) membrane | In situ synthesis and assembly | Surfactant-stabilized oil/water emulsions | Pressure-driven (N₂, 1.0 bar) | NR | 5762.92 | Concentration-based rejection efficiency | 96.3 |
| L12[12] | PS/PAN-PVDF/PDMS@TiO₂ composite membrane | Electrospinning | n-Hexane/water mixture | Gravity-driven | NR | 6000 | Volumetric oil recovery efficiency | 97 |
| L13[13] | Janus membrane (PVDF/Ca₁₀(PO₄)₆(OH)₂@PET) | Impregnation method + Electrospinning | Oil/water mixtures (CCl₄, 1,2-dichloroethane, toluene, n-hexadecane, diesel oil, gasoline) | Gravity-driven | 2 × 2 cm² (effective area: 4 cm²) | 153.29–324.27 | Mass-based recovery efficiency | >90 |
| This work | Janus-like RCM | NIPS | Water/peanut oil, water/n-hexane, water/DCM mixtures | Gravity-driven (without external pressure) | 4.3 × 4.5 cm (effective area: 19.35 cm²) | 3300.83–5527.68 | Apparent volumetric oil-retention efficiency | 95–96.93 (oil/water systems); 70.14 (DCM/water) |

**Reference**

[1]	M. Zhu, L. Zhang, H. Ma, R. Zhu, Y. Tan, R. Wang, X. Gong, Y. Liu, Janus stainless steel mesh-based membrane with asymmetric wettability for highly efficient gravity-driven oil-water emulsion separation, Journal of Membrane Science 702 (2024) 122801. https://doi.org/10.1016/j.memsci.2024.122801.

[2]	W. Zhu, W. Zhu, Z. Xu, Y. Song, H. Cen, S. Cao, Y. Huang, Y. Li, A facile lamination strategy for constructing Janus membranes with adjustable thickness for humidity management and oil–water separation, Separation and Purification Technology 365 (2025) 132676. https://doi.org/10.1016/j.seppur.2025.132676.

[3]	K. Chen, J. Zhu, C. Hao, H. Zhang, Y. Tan, X. Xiao, F. Sun, X. Han, H. Peng, T. Zhan, J. Lyu, N. Yan, Integrated emulsion separation and fog collection with functionalized Janus wood membrane for water scarcity solutions, Npj Clean Water 8 (2025) 1–11. https://doi.org/10.1038/s41545-025-00460-8.

[4]	R. Zhang, X. Ren, P. Cai, L. Zhong, Y. Lu, Z. Zhou, Designing integrated Janus membrane based on a double-cross-linked polyphenol hydrophilic layer for complex emulsion separation, Separation and Purification Technology 330 (2024) 125417. https://doi.org/10.1016/j.seppur.2023.125417.

[5]	Y. Zhang, Y. Li, B. Huang, Y. Chen, Y. Xu, J. Liu, K. Liu, Y. Zeng, Bioinspired fibrous Murray membrane with gradual-transition wettability and pore size for directional water transport and oil/water emulsion separation, Separation and Purification Technology 372 (2025) 133508. https://doi.org/10.1016/j.seppur.2025.133508.

[6]	K. Li, G. Feng, G. Li, Z. Zhang, J. Xiang, F. Jiao, T. Chen, H. Zhao, Structurally integrated janus polylactic acid fibrous membranes for oil-water separation, Journal of Water Process Engineering 68 (2024) 106525. https://doi.org/10.1016/j.jwpe.2024.106525.

[7]	H. Wu, J. Shi, X. Ning, Y.-Z. Long, J. Zheng, The High Flux of Superhydrophilic-Superhydrophobic Janus Membrane of cPVA-PVDF/PMMA/GO by Layer-by-Layer Electrospinning for High Efficiency Oil-Water Separation, Polymers 14 (2022) 621. https://doi.org/10.3390/polym14030621.

[8]	X. Chen, Y. Yang, Y. Li, M. Bao, D. Zhang, Z. Wang, Integrated asymmetric superwetting Janus membrane for the efficient separation of various surfactant-stabilized oil–water emulsions, Environ. Sci.: Nano 8 (2021) 2235–2248. https://doi.org/10.1039/D1EN00335F.

[9]	H. Lin, F. Yu, J. Liang, S. Bai, R. Cai, P. Xu, P. Zhang, Y. Luo, Biodegradable Janus membrane for oil/water separation and unidirectional oil transport via electrospinning and plasma treatment, Journal of Cleaner Production 525 (2025) 146514. https://doi.org/10.1016/j.jclepro.2025.146514.

[10]	W. Zhu, Y. Xing, H. Wang, M. Yu, H. Cen, L. Liu, Y. Li, W. Zhu, Fluorine-free preparation of superhydrophobic polyester fabric with directional moisture transport for efficient oil-water separation, Colloids and Surfaces A: Physicochemical and Engineering Aspects 696 (2024) 134369. https://doi.org/10.1016/j.colsurfa.2024.134369.

[11]	L. Fei, L. Shen, C. Chen, J. Xu, B. Wang, B. Li, H. Lin, Assembling 99% MOFs into Bioinspired Rigid-Flexible Coupled Membrane with Significant Permeability: The Impacts of Defects, Small 20 (2024) 2306528. https://doi.org/10.1002/smll.202306528.

[12]	X. Wang, X. Li, Preparation of superhydrophobic membranes with ultraviolet-absorbing capacity for oil–water separation by electrospinning, Polymer Engineering & Science 64 (2024) 2035–2048. https://doi.org/10.1002/pen.26670.

[13]	B. Tian, B. Ren, M. Hu, Y. Yang, J. Wu, Janus membrane with asymmetric wettability for efficient oil/water separation, Heliyon 9 (2023) e22534. https://doi.org/10.1016/j.heliyon.2023.e22534.
